# Bis{4,4′-[oxalylbis(aza­nedi­yl)]dipyridinium} octa­molybdate

**DOI:** 10.1107/S1600536810020714

**Published:** 2010-06-05

**Authors:** Jianbo Qin, Jingren Dong, Jinghua Li, Yun Gong

**Affiliations:** a Department of Chemistry, College of Chemistry and Chemical Engineering, Chongqing University 400044, People’s Republic of China; b Department of Pharmaceutical Chemistry, College of Chemistry and Chemical Engineering, Chongqing University 400044, People’s Republic of China

## Abstract

In the crystal structure of the title compound, (C_12_H_12_N_4_O_2_)_2_[Mo_8_O_26_], the amino and pyridinium groups of the *N* ^1^,*N* ^2^-di(pyridinium-4-yl)oxalamide cations are hydrogen bonded to the O atoms of the centrosymmetric isopolyoxometalate β-[Mo_8_O_26_]^4−^ anions, forming a three-dimensional supra­molecular architecture.

## Related literature

For polyoxometalates (POMs), see: Cronin *et al.* (2002 ▶); Fukaya & Yamase (2003 ▶); Katsoulis (1988 ▶); Pope & Müller (1991 ▶). For the applications of POMs in biology and materials sciences, see: Cui *et al.* (2003 ▶); Luan *et al.* (2002 ▶); Wang *et al.* (2003 ▶). For the structure of *N* ^1^,*N* ^2^-di(pyridin-4-yl)oxalamide, see: Tzeng *et al.* (2007 ▶). For details of the geometrical parameters in the same isopolyoxometalate anion, see: Gong *et al.* (2007 ▶).
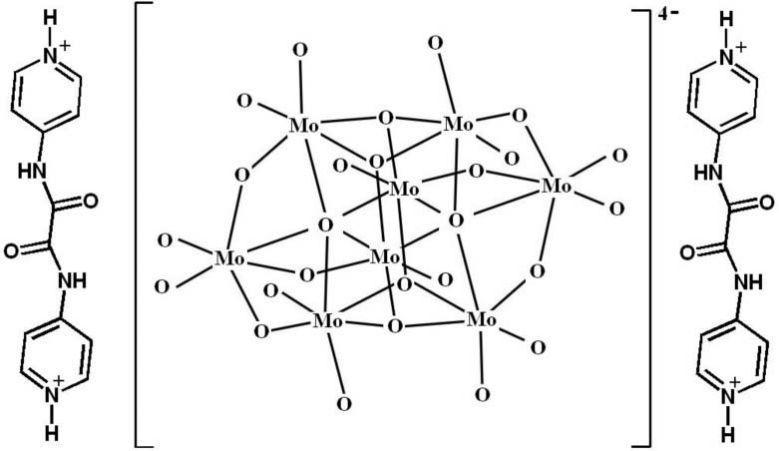


## Experimental

### 

#### Crystal data

(C_12_H_12_N_4_O_2_)_2_[Mo_8_O_26_]
*M* *_r_* = 1672.03
Monoclinic, 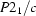

*a* = 10.633 (2) Å
*b* = 11.552 (2) Å
*c* = 17.240 (4) Å
β = 101.553 (3)°
*V* = 2074.7 (8) Å^3^
*Z* = 2
Mo *K*α radiation
μ = 2.45 mm^−1^
*T* = 293 K
0.23 × 0.22 × 0.05 mm

#### Data collection

Siemens SMART CCD area-detector diffractometer
Absorption correction: multi-scan (*SADABS*; Sheldrick, 1996 ▶) *T*_min_ = 0.829, *T*_max_ = 1.000
14669 measured reflections
4534 independent reflections
4215 reflections with *I* > 2σ(*I*)
*R*_int_ = 0.021

#### Refinement

*R*[*F* ^2^ > 2σ(*F* ^2^)] = 0.022
*wR*(*F* ^2^) = 0.098
*S* = 1.40
4534 reflections
316 parameters
H-atom parameters constrained
Δρ_max_ = 1.40 e Å^−3^
Δρ_min_ = −2.27 e Å^−3^

### 

Data collection: *SMART* (Siemens, 1996 ▶); cell refinement: *SAINT* (Siemens, 1996 ▶); data reduction: *SAINT*; program(s) used to solve structure: *SHELXS97* (Sheldrick, 2008 ▶); program(s) used to refine structure: *SHELXL97* (Sheldrick, 2008 ▶); molecular graphics: *SHELXTL* (Sheldrick, 2008 ▶); software used to prepare material for publication: *SHELXTL*.

## Supplementary Material

Crystal structure: contains datablocks global, I. DOI: 10.1107/S1600536810020714/su2182sup1.cif

Structure factors: contains datablocks I. DOI: 10.1107/S1600536810020714/su2182Isup2.hkl

Additional supplementary materials:  crystallographic information; 3D view; checkCIF report

## Figures and Tables

**Table 1:** Hydrogen-bond geometry (Å, °)

*D*—H⋯*A*	*D*—H	H⋯*A*	*D*⋯*A*	*D*—H⋯*A*
N2—H2*A*⋯O7^i^	0.86	2.61	3.368 (4)	148
N1—H1⋯O8^ii^	0.86	1.89	2.699 (4)	158
N3—H3⋯O5^iii^	0.86	1.94	2.779 (4)	165
N4—H4*A*⋯O1	0.86	2.25	2.669 (4)	110
N4—H4*A*⋯O4^iv^	0.86	2.26	3.059 (4)	154

Symmetry codes: (i) 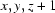; (ii) 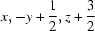; (iii) 
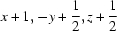
; (iv) 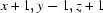.

**Fig. 1.**
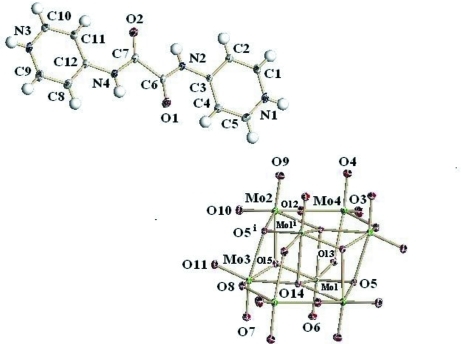
The molecular structure of the title complex, with the atomic numbering scheme and displacement ellipsoids at the 30% probability level [Symmetry codes: (i) -x,-y+1,-z].

**Fig. 2.**
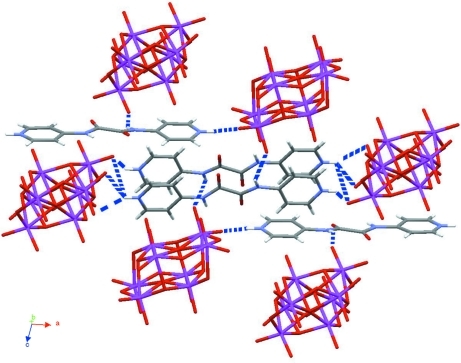
A view along the b-axis of the crystal packing of the title complex, illustrating the three dimensional supramolecular architecture constructed by the intermolecular N-H···O hydrogen bonds (dotted lines); see Table 1 for details.

## supplementary crystallographic information

### Comment

Polyoxometalates (POMs) are early transition metal oxygen anion clusters. They are an outstanding class of anionic compounds due to their wealthy topology, superior physical and chemical properties (Pope & Muller, 1991; Katsoulis, 1988). The nanoscopic sizes (Cronin, *et al.*, 2002; Fukaya & Yamase, 2003,) and thier diversified shapes of discrete POMs have attracted great interest. The design, synthesis and structural characterization of inorganic-organic hybrid compounds base on POMs, for which many properties and applications can be predicted, have established a new field of research in the chemistry of biology and materials sciences (Luan, *et al.*, 2002; Cui, *et al.*, 2003; Wang, *et al.*, 2003). Different N-heterocycle ligands can lead to different inorganic-organic hybrid compounds based on POMs. N^1^,N^2^-di(pyridin-4-yl)oxalamide (L), is a bis-pyridine ligand, which has been reported only rarely in the construction of hybrid compounds based on POMs. In the present work, the title complex was synthesized hydrothermally by reacting L with the isopolyoxometalate, Mo_8_O_26_.

The molecular structure of the title complex is illustrated in Fig. 1. In the asymmetric unit there is a doublely protonated L molecule, and half an isopolyoxometalate unit. The bond distances and angles in the cation are similar to those observed previously for N^1^,N^2^-di(pyridin-4-yl)oxalamide (Tzeng, *et al.*, 2007). For the anion, [Mo_8_O_26_]^4-^, the geometrical parameters are similar to those reported by (Gong, *et al.*, 2007).

In the crystal the protonated pyrdidinium groups and the amino group form N-H···O hydrogen bonds with the oxygen atoms of the centrosymmetric [Mo_8_O_26_]^4-^ anions, leading to the formation of a three dimensional supramolecular network (Table 1 and Fig. 2).

### Experimental

A mixture of L (0.05 mmol, 0.012 g), Na_2_MoO_4_(0.05 mmol, 0.012 g) and water(10 ml) was adjusted to pH = 3.0 by HCl. The synthesis was carried out hydrothermally using a Teflon-lined autoclave. The reaction mixture was heated at 393 K for 3 days, followed by slow cooling to rt. The resulting colorless prismatic crystals were filtered off and washed with water (yield: ca. 90% based on Mo). Elemental analyse - found: C, 17.45; H, 1.58; N, 6.56; Mo, 46.11; calcd: C, 17.22; H, 1.44; N, 6.70; Mo, 45.93.

### Refinement

The H-atoms were positioned geometrically and refined as riding atoms: C—H = 0.93Å, N—H = 0.86Å and *U*_iso_(H) = 1.2*U*_eq_(N,C).

### Crystal data

**Table:**

(C_12_H_12_N_4_O_2_)_2_[Mo_8_O_26_]	*F*(000) = 1592
*M**_r_* = 1672.03	*D*_x_ = 2.670 Mg m^−^^3^
Monoclinic, *P*2_1_/*c*	Mo *K*α radiation, λ = 0.71073 Å
Hall symbol: -P 2ybc	Cell parameters from 5569 reflections
*a* = 10.633 (2) Å	θ = 2.1–27.5°
*b* = 11.552 (2) Å	µ = 2.45 mm^−^^1^
*c* = 17.240 (4) Å	*T* = 293 K
β = 101.553 (3)°	Prism, colorless
*V* = 2074.7 (8) Å^3^	0.23 × 0.22 × 0.05 mm
*Z* = 2	

### Data collection

**Table:**

Siemens CCD area-detector diffractometer	4534 independent reflections
Radiation source: fine-focus sealed tube	4215 reflections with *I* > 2σ(*I*)
graphite	*R*_int_ = 0.021
φ and ω scans	θ_max_ = 27.5°, θ_min_ = 2.1°
Absorption correction: multi-scan (*SADABS*; Sheldrick, 1996)	*h* = −13→13
*T*_min_ = 0.829, *T*_max_ = 1.000	*k* = −13→14
14669 measured reflections	*l* = −22→17

### Refinement

**Table:**

Refinement on *F*^2^	Primary atom site location: structure-invariant direct methods
Least-squares matrix: full	Secondary atom site location: difference Fourier map
*R*[*F*^2^ > 2σ(*F*^2^)] = 0.022	Hydrogen site location: inferred from neighbouring sites
*wR*(*F*^2^) = 0.098	H-atom parameters constrained
*S* = 1.40	*w* = 1/[σ^2^(*F*_o_^2^) + (0.054*P*)^2^ + 0.4946*P*] where *P* = (*F*_o_^2^ + 2*F*_c_^2^)/3
4534 reflections	(Δ/σ)_max_ = 0.001
316 parameters	Δρ_max_ = 1.40 e Å^−^^3^
0 restraints	Δρ_min_ = −2.27 e Å^−^^3^

### Special details

**Table:**

Geometry. All esds (except the esd in the dihedral angle between two l.s. planes) are estimated using the full covariance matrix. The cell esds are taken into account individually in the estimation of esds in distances, angles and torsion angles; correlations between esds in cell parameters are only used when they are defined by crystal symmetry. An approximate (isotropic) treatment of cell esds is used for estimating esds involving l.s. planes.
Refinement. Refinement of F^2^ against ALL reflections. The weighted R-factor wR and goodness of fit S are based on F^2^, conventional R-factors R are based on F, with F set to zero for negative F^2^. The threshold expression of F^2^ > 2sigma(F^2^) is used only for calculating R-factors(gt) etc. and is not relevant to the choice of reflections for refinement. R-factors based on F^2^ are statistically about twice as large as those based on F, and R- factors based on ALL data will be even larger.

### Fractional atomic coordinates and isotropic or equivalent isotropic displacement parameters (Å^2^)

**Table:**

	*x*	*y*	*z*	*U*_iso_*/*U*_eq_	
Mo1	0.08119 (2)	0.53770 (2)	0.098624 (15)	0.01481 (10)	
Mo2	0.03951 (3)	0.71904 (2)	−0.069243 (16)	0.01713 (10)	
Mo3	0.25513 (2)	0.49409 (2)	−0.029720 (16)	0.01602 (10)	
Mo4	−0.13294 (3)	0.77083 (2)	0.064661 (17)	0.01917 (10)	
O12	0.0104 (2)	0.8099 (2)	0.01704 (14)	0.0218 (5)	
O6	0.2011 (2)	0.4893 (2)	0.17096 (15)	0.0252 (5)	
O15	0.1779 (2)	0.61483 (18)	0.02814 (13)	0.0168 (4)	
O5	−0.0636 (2)	0.44017 (19)	0.11312 (13)	0.0165 (4)	
O4	−0.2513 (2)	0.8238 (2)	−0.00841 (16)	0.0297 (6)	
O7	0.3709 (2)	0.4459 (2)	0.04628 (15)	0.0275 (5)	
N2	0.5229 (3)	0.1925 (2)	1.09498 (17)	0.0221 (6)	
H2A	0.5102	0.2587	1.0720	0.026*	
O13	0.0261 (2)	0.6628 (2)	0.13799 (13)	0.0205 (5)	
C2	0.3935 (3)	0.2606 (3)	1.1832 (2)	0.0259 (7)	
H2	0.3675	0.3224	1.1491	0.031*	
O11	0.3343 (2)	0.5745 (2)	−0.08718 (14)	0.0242 (5)	
O9	−0.0852 (3)	0.7563 (2)	−0.14308 (15)	0.0274 (5)	
O10	0.1691 (3)	0.7832 (2)	−0.09476 (16)	0.0273 (5)	
C5	0.4718 (4)	0.0833 (3)	1.2863 (2)	0.0301 (8)	
H5	0.4985	0.0245	1.3227	0.036*	
C4	0.5210 (4)	0.0892 (3)	1.2190 (2)	0.0265 (7)	
H4	0.5801	0.0346	1.2091	0.032*	
N1	0.3858 (3)	0.1609 (3)	1.30054 (17)	0.0280 (7)	
H1	0.3545	0.1541	1.3426	0.034*	
O3	−0.1253 (3)	0.8654 (2)	0.14042 (16)	0.0321 (6)	
C3	0.4805 (3)	0.1793 (3)	1.16520 (18)	0.0196 (6)	
C1	0.3471 (4)	0.2490 (3)	1.2508 (2)	0.0286 (8)	
H1A	0.2882	0.3025	1.2626	0.034*	
O14	0.0844 (2)	0.40727 (19)	0.01120 (12)	0.0173 (4)	
O8	0.2273 (2)	0.3590 (2)	−0.09586 (13)	0.0205 (5)	
N3	0.8440 (3)	0.0969 (3)	0.75088 (17)	0.0285 (7)	
H3	0.8761	0.0988	0.7089	0.034*	
O2	0.5624 (3)	0.2419 (2)	0.94778 (15)	0.0289 (6)	
O1	0.6119 (3)	0.0145 (2)	1.08260 (16)	0.0334 (6)	
N4	0.6865 (3)	0.0776 (3)	0.94989 (17)	0.0236 (6)	
H4A	0.7065	0.0154	0.9770	0.028*	
C11	0.7132 (3)	0.1824 (3)	0.8303 (2)	0.0261 (7)	
H11	0.6606	0.2428	0.8399	0.031*	
C10	0.7680 (4)	0.1841 (3)	0.7648 (2)	0.0298 (8)	
H10	0.7525	0.2461	0.7297	0.036*	
C8	0.8193 (4)	0.0012 (3)	0.8668 (2)	0.0303 (8)	
H8	0.8384	−0.0609	0.9015	0.036*	
C9	0.8712 (4)	0.0072 (3)	0.8005 (2)	0.0333 (9)	
H9	0.9256	−0.0512	0.7898	0.040*	
C12	0.7373 (3)	0.0892 (3)	0.88212 (18)	0.0204 (6)	
C6	0.5830 (3)	0.1107 (3)	1.05882 (19)	0.0206 (6)	
C7	0.6084 (3)	0.1539 (3)	0.97837 (19)	0.0226 (7)	

### Atomic displacement parameters (Å^2^)

**Table:**

	*U*^11^	*U*^22^	*U*^33^	*U*^12^	*U*^13^	*U*^23^
Mo1	0.01333 (15)	0.02061 (16)	0.01084 (15)	−0.00116 (9)	0.00325 (10)	−0.00091 (10)
Mo2	0.01807 (16)	0.01867 (16)	0.01602 (16)	0.00122 (10)	0.00673 (11)	0.00088 (10)
Mo3	0.01266 (15)	0.02166 (17)	0.01468 (16)	0.00069 (9)	0.00496 (11)	0.00006 (10)
Mo4	0.01891 (17)	0.02088 (16)	0.01936 (16)	0.00103 (10)	0.00773 (12)	−0.00038 (10)
O12	0.0232 (12)	0.0214 (11)	0.0228 (11)	−0.0027 (9)	0.0093 (10)	−0.0038 (9)
O6	0.0216 (12)	0.0342 (13)	0.0184 (12)	0.0020 (10)	0.0003 (10)	0.0029 (10)
O15	0.0156 (10)	0.0194 (10)	0.0160 (10)	−0.0018 (8)	0.0047 (8)	−0.0007 (9)
O5	0.0162 (10)	0.0199 (10)	0.0146 (10)	0.0006 (8)	0.0057 (8)	0.0005 (9)
O4	0.0250 (13)	0.0339 (14)	0.0311 (14)	0.0052 (11)	0.0073 (11)	0.0089 (11)
O7	0.0216 (12)	0.0381 (14)	0.0221 (12)	0.0048 (11)	0.0026 (10)	0.0052 (11)
N2	0.0260 (15)	0.0251 (14)	0.0176 (13)	0.0021 (12)	0.0105 (11)	0.0042 (11)
O13	0.0204 (11)	0.0226 (11)	0.0192 (11)	−0.0004 (9)	0.0059 (9)	−0.0040 (9)
C2	0.0268 (18)	0.0300 (17)	0.0226 (17)	0.0055 (14)	0.0089 (14)	0.0044 (14)
O11	0.0217 (12)	0.0292 (12)	0.0237 (12)	−0.0022 (10)	0.0091 (10)	0.0032 (10)
O9	0.0291 (13)	0.0281 (12)	0.0232 (12)	0.0072 (11)	0.0010 (11)	0.0008 (11)
O10	0.0272 (13)	0.0269 (13)	0.0305 (13)	−0.0026 (10)	0.0127 (11)	0.0019 (10)
C5	0.038 (2)	0.0303 (18)	0.0222 (17)	0.0010 (16)	0.0069 (15)	0.0068 (15)
C4	0.0269 (18)	0.0316 (18)	0.0222 (17)	0.0038 (14)	0.0074 (14)	0.0026 (15)
N1	0.0295 (16)	0.0401 (17)	0.0171 (13)	−0.0029 (13)	0.0114 (12)	−0.0004 (13)
O3	0.0354 (14)	0.0310 (13)	0.0332 (14)	0.0015 (11)	0.0146 (12)	−0.0097 (12)
C3	0.0180 (15)	0.0268 (16)	0.0148 (14)	−0.0016 (12)	0.0053 (12)	0.0005 (13)
C1	0.0263 (18)	0.0380 (19)	0.0235 (17)	0.0048 (15)	0.0097 (15)	−0.0007 (16)
O14	0.0169 (10)	0.0209 (10)	0.0153 (10)	0.0003 (8)	0.0063 (8)	0.0001 (9)
O8	0.0223 (11)	0.0228 (11)	0.0193 (11)	0.0002 (9)	0.0110 (9)	−0.0029 (9)
N3	0.0311 (16)	0.0405 (17)	0.0173 (13)	−0.0040 (14)	0.0130 (12)	−0.0015 (13)
O2	0.0333 (14)	0.0309 (13)	0.0244 (13)	0.0054 (11)	0.0104 (11)	0.0031 (11)
O1	0.0461 (17)	0.0300 (14)	0.0293 (14)	0.0072 (12)	0.0204 (13)	0.0039 (11)
N4	0.0278 (15)	0.0276 (14)	0.0188 (13)	0.0042 (12)	0.0127 (12)	0.0029 (12)
C11	0.0261 (17)	0.0322 (18)	0.0218 (16)	0.0051 (14)	0.0090 (14)	0.0019 (14)
C10	0.0299 (19)	0.040 (2)	0.0209 (17)	0.0027 (16)	0.0074 (15)	0.0061 (15)
C8	0.040 (2)	0.0269 (18)	0.0284 (19)	0.0052 (15)	0.0175 (17)	0.0041 (15)
C9	0.042 (2)	0.0317 (19)	0.032 (2)	−0.0007 (16)	0.0210 (18)	−0.0033 (16)
C12	0.0232 (16)	0.0244 (16)	0.0155 (14)	−0.0036 (13)	0.0081 (12)	−0.0026 (12)
C6	0.0195 (15)	0.0278 (16)	0.0171 (14)	−0.0042 (13)	0.0094 (12)	−0.0017 (13)
C7	0.0235 (16)	0.0286 (16)	0.0172 (15)	−0.0049 (14)	0.0076 (13)	−0.0018 (14)

### Geometric parameters (Å, °)

**Table:**

Mo1—O6	1.690 (2)	C2—C3	1.395 (5)
Mo1—O13	1.747 (2)	C2—H2	0.9300
Mo1—O15	1.956 (2)	C5—N1	1.338 (5)
Mo1—O5	1.964 (2)	C5—C4	1.366 (5)
Mo1—O14	2.136 (2)	C5—H5	0.9300
Mo1—O14^i^	2.399 (2)	C4—C3	1.403 (5)
Mo1—Mo3	3.1951 (6)	C4—H4	0.9300
Mo2—O10	1.699 (3)	N1—C1	1.342 (5)
Mo2—O9	1.699 (3)	N1—H1	0.8600
Mo2—O12	1.896 (2)	C1—H1A	0.9300
Mo2—O5^i^	2.024 (2)	O14—Mo2^i^	2.322 (2)
Mo2—O14^i^	2.322 (2)	O14—Mo1^i^	2.399 (2)
Mo2—O15	2.333 (2)	O8—Mo4^i^	1.939 (2)
Mo3—O11	1.699 (2)	N3—C9	1.338 (5)
Mo3—O7	1.702 (2)	N3—C10	1.342 (5)
Mo3—O8	1.921 (2)	N3—H3	0.8600
Mo3—O15	1.986 (2)	O2—C7	1.203 (4)
Mo3—O14	2.305 (2)	O1—C6	1.203 (4)
Mo3—O5^i^	2.370 (2)	N4—C7	1.368 (4)
Mo4—O3	1.692 (3)	N4—C12	1.388 (4)
Mo4—O4	1.706 (3)	N4—H4A	0.8600
Mo4—O12	1.924 (2)	C11—C10	1.371 (5)
Mo4—O8^i^	1.939 (2)	C11—C12	1.390 (5)
Mo4—O13	2.271 (2)	C11—H11	0.9300
O5—Mo2^i^	2.024 (2)	C10—H10	0.9300
O5—Mo3^i^	2.370 (2)	C8—C9	1.368 (5)
N2—C6	1.360 (4)	C8—C12	1.398 (5)
N2—C3	1.383 (4)	C8—H8	0.9300
N2—H2A	0.8600	C9—H9	0.9300
C2—C1	1.361 (5)	C6—C7	1.548 (5)
			
O6—Mo1—O13	104.42 (12)	O12—Mo4—O13	78.54 (10)
O6—Mo1—O15	101.34 (11)	O8^i^—Mo4—O13	77.86 (9)
O13—Mo1—O15	97.08 (10)	Mo2—O12—Mo4	118.21 (12)
O6—Mo1—O5	102.18 (11)	Mo1—O15—Mo3	108.29 (10)
O13—Mo1—O5	95.32 (10)	Mo1—O15—Mo2	110.37 (10)
O15—Mo1—O5	149.67 (9)	Mo3—O15—Mo2	105.41 (9)
O6—Mo1—O14	99.85 (11)	Mo1—O5—Mo2^i^	108.21 (10)
O13—Mo1—O14	155.72 (10)	Mo1—O5—Mo3^i^	109.83 (10)
O15—Mo1—O14	78.37 (9)	Mo2^i^—O5—Mo3^i^	102.84 (9)
O5—Mo1—O14	78.98 (9)	C6—N2—C3	126.2 (3)
O6—Mo1—O14^i^	174.94 (10)	C6—N2—H2A	116.9
O13—Mo1—O14^i^	80.62 (9)	C3—N2—H2A	116.9
O15—Mo1—O14^i^	77.46 (8)	Mo1—O13—Mo4	120.57 (11)
O5—Mo1—O14^i^	77.42 (8)	C1—C2—C3	119.6 (3)
O14—Mo1—O14^i^	75.11 (9)	C1—C2—H2	120.2
O6—Mo1—Mo3	90.19 (9)	C3—C2—H2	120.2
O13—Mo1—Mo3	133.25 (8)	N1—C5—C4	121.0 (3)
O15—Mo1—Mo3	36.17 (6)	N1—C5—H5	119.5
O5—Mo1—Mo3	125.10 (6)	C4—C5—H5	119.5
O14—Mo1—Mo3	46.13 (6)	C5—C4—C3	118.6 (3)
O14^i^—Mo1—Mo3	86.01 (5)	C5—C4—H4	120.7
O10—Mo2—O9	104.31 (13)	C3—C4—H4	120.7
O10—Mo2—O12	103.29 (11)	C5—N1—C1	121.6 (3)
O9—Mo2—O12	102.84 (11)	C5—N1—H1	119.2
O10—Mo2—O5^i^	97.46 (11)	C1—N1—H1	119.2
O9—Mo2—O5^i^	95.06 (11)	N2—C3—C2	117.9 (3)
O12—Mo2—O5^i^	148.09 (10)	N2—C3—C4	123.3 (3)
O10—Mo2—O14^i^	160.99 (11)	C2—C3—C4	118.8 (3)
O9—Mo2—O14^i^	93.28 (11)	N1—C1—C2	120.3 (3)
O12—Mo2—O14^i^	79.28 (9)	N1—C1—H1A	119.8
O5^i^—Mo2—O14^i^	73.47 (8)	C2—C1—H1A	119.8
O10—Mo2—O15	89.10 (11)	Mo1—O14—Mo3	91.94 (8)
O9—Mo2—O15	162.69 (11)	Mo1—O14—Mo2^i^	92.79 (8)
O12—Mo2—O15	84.24 (9)	Mo3—O14—Mo2^i^	162.71 (11)
O5^i^—Mo2—O15	72.02 (8)	Mo1—O14—Mo1^i^	104.89 (9)
O14^i^—Mo2—O15	72.31 (8)	Mo3—O14—Mo1^i^	98.15 (8)
O11—Mo3—O7	105.16 (12)	Mo2^i^—O14—Mo1^i^	96.69 (8)
O11—Mo3—O8	97.69 (11)	Mo3—O8—Mo4^i^	119.38 (11)
O7—Mo3—O8	101.11 (12)	C9—N3—C10	121.9 (3)
O11—Mo3—O15	102.23 (11)	C9—N3—H3	119.1
O7—Mo3—O15	98.71 (11)	C10—N3—H3	119.1
O8—Mo3—O15	147.03 (9)	C7—N4—C12	127.3 (3)
O11—Mo3—O14	158.28 (10)	C7—N4—H4A	116.3
O7—Mo3—O14	96.57 (11)	C12—N4—H4A	116.3
O8—Mo3—O14	77.93 (9)	C10—C11—C12	119.1 (3)
O15—Mo3—O14	73.83 (8)	C10—C11—H11	120.4
O11—Mo3—O5^i^	86.39 (10)	C12—C11—H11	120.4
O7—Mo3—O5^i^	166.64 (10)	N3—C10—C11	120.4 (3)
O8—Mo3—O5^i^	83.62 (9)	N3—C10—H10	119.8
O15—Mo3—O5^i^	71.82 (8)	C11—C10—H10	119.8
O14—Mo3—O5^i^	72.03 (8)	C9—C8—C12	119.5 (3)
O11—Mo3—Mo1	137.78 (9)	C9—C8—H8	120.3
O7—Mo3—Mo1	87.02 (9)	C12—C8—H8	120.3
O8—Mo3—Mo1	119.84 (7)	N3—C9—C8	120.1 (4)
O15—Mo3—Mo1	35.55 (6)	N3—C9—H9	119.9
O14—Mo3—Mo1	41.93 (5)	C8—C9—H9	119.9
O5^i^—Mo3—Mo1	79.83 (5)	N4—C12—C11	124.3 (3)
O3—Mo4—O4	104.63 (14)	N4—C12—C8	116.8 (3)
O3—Mo4—O12	104.98 (12)	C11—C12—C8	118.9 (3)
O4—Mo4—O12	97.54 (12)	O1—C6—N2	126.7 (3)
O3—Mo4—O8^i^	103.44 (11)	O1—C6—C7	121.6 (3)
O4—Mo4—O8^i^	97.81 (12)	N2—C6—C7	111.6 (3)
O12—Mo4—O8^i^	142.92 (9)	O2—C7—N4	127.6 (3)
O3—Mo4—O13	90.56 (11)	O2—C7—C6	122.5 (3)
O4—Mo4—O13	164.80 (11)	N4—C7—C6	110.0 (3)

Symmetry codes: (i) −*x*, −*y*+1, −*z*.

### Hydrogen-bond geometry (Å, °)

**Table:**

*D*—H···*A*	*D*—H	H···*A*	*D*···*A*	*D*—H···*A*
N2—H2A···O7^ii^	0.86	2.61	3.368 (4)	148
N1—H1···O8^iii^	0.86	1.89	2.699 (4)	158
N3—H3···O5^iv^	0.86	1.94	2.779 (4)	165
N4—H4A···O1	0.86	2.25	2.669 (4)	110
N4—H4A···O4^v^	0.86	2.26	3.059 (4)	154

Symmetry codes: (ii) *x*, *y*, *z*+1; (iii) *x*, −*y*+1/2, *z*+3/2; (iv) *x*+1, −*y*+1/2, *z*+1/2;  (v) *x*+1, *y*−1, *z*+1.
